# Efficacy of once or twice weekly administration of epoetin κ in patients receiving hemodialysis: A retrospective study

**DOI:** 10.3892/etm.2013.1384

**Published:** 2013-11-06

**Authors:** SHOICHIRO OHTA, NOBUHIRO YASUNO, YUKI INOMOTO, KAORI MATSUDA, YOSHIHIKO NAKAGAWA, ISOJI SASAGAWA, MASAHIKO TANAKA

**Affiliations:** 1 Department of Urology and Hemodialysis, Kan-Etsu Hospital, Tsurugashima, Saitama, Japan; 2 Department of Pharmacy, Kan-Etsu Hospital, Tsurugashima, Saitama, Japan; 3 Minami-Cho Clinic, Sakado, Saitama, Japan; 4 Department of Urology, Yamagata Tokushukai Hospital, Yamagata, Japan; 5 Department of Rheumatology, Kan-Etsu Hospital, Tsurugashima, Saitama, Japan

**Keywords:** epoetin-κ, epoetin-α, biosimilar, hemodialysis, renal failure

## Abstract

Several clinically approved recombinant erythropoietin (rEPO) preparations, such as epoetin-β, epoetin-δ and the epoetin-α derivative, darbepoetin-α, have been commercially produced. Since the expiration of patent protection, a number of novel rEPO biosimilars have been approved on the world market. In 2010, epoetin-κ, which is biosimilar to epoetin-α, was clinically approved. Epoetin-κ is a biopharmaceutical product that is based on serum-free media following master cell bank preparation. The present study analyzes the results obtained during a six-month observation period, in which the administration of epoetin-β was switched to that of epoetin-κ. In a cohort of patients receiving chronic dialysis, who were clinically in a state of relative calm and were in control of their renal anemia, it was possible to sustain good control of the anemia by reducing the frequency of the epoetin-β administration from the conventional and empirically determined three times a week to twice a week, and further to once a week. Furthermore, the good control was maintained upon changing from the administration of epoetin-β to that of epoetin-κ. Moreover, three months subsequent to this switch, the degree of instability observed among the patients had decreased. Despite the fact that the situation following the changeover requires further investigation, it may be concluded that the results obtained in this study are indicative of the clinical equivalence and efficacy of epoetin-κ.

## Introduction

Erythropoietin (EPO) is a hormone that is predominantly produced by the kidneys. It acts on the bone marrow to stimulate the proliferation and differentiation of red blood cells. In 1989, the first recombinant EPO (rEPO) preparation, epoetin-α, was approved by the US Food and Drug Administration for the treatment of anemia associated with kidney disease (1,2). Since then, several clinically approved rEPO preparations, such as epoetin-β, epoetin-δ and the epoetin-α derivative, darbepoietin-α, have been commercially produced. Since the expiration of patent protection, a number of novel rEPO biosimilars have been approved on the world market. In 2010, epoetin-κ, which is biosimilar to epoetin-α, was clinically approved in Japan (3). Epoetin-κ is a biopharmaceutical product that is based on serum-free media following master cell bank preparation. In general, and as is the case with this medicine, the manufacture of treatments based on genetic recombination that utilize animal cells requires the use of adhesive cells to function as the serum constituent (4). As a result, it is not easy to obtain cells with steady levels of productivity from a non-blood serum that is created only from a general culture medium (2–4). However, the preparation for epoetin-κ involves a new manufacturing method, being cultivated using a bloodless liquid medium and a purification process that does not utilize animal byproducts. It has been indicated that epoetin-κ exhibits the same efficacy as a preparation of erythropoietin, which, to date, has been used with a basic three-times-a-week administration, resulting in the demonstration of its safety and quality (5–7).

The present study examined a cohort of patients receiving chronic dialysis who were clinically in a state of relative calm and whose renal anemia was well controlled. The ability to sustain good control of the anemia when the frequency of the epoetin-β administration was reduced from the conventional and empirically determined three times a week to twice a week, and further to once a week (8,9) was assessed. Furthermore, the ability to maintain good control upon switching from epoetin-β to epoetin-κ was investigated. The impact of the change, including the economic effect, was also considered.

## Patients and methods

Thirty patients were maintained at The Kan-Etsu Hospital (Tsurugashima, Japan) on dialysis, with an epoetin dosing frequency of once or twice a week. The clinical parameters of the patients that were associated with anemia (red blood cell counts, hemaglobin and hematocrit) were obtained over a six-month period, during which the administration of epoetin-β was changed to that of epoetin-κ. The clinical parameters collected in the three months prior to and following the switch, respectively, were subsequently compared. Males accounted for a large proportion of the patients (26) and 16 of those males were aged ≥65 years (Table I). There were eight patients with underlying diabetes (Table I). The frequency of drug administration for the 30 patients was twice a week or less. Monthly blood samples were obtained from the patients in the three months prior to and following the changeover from epoetin-β to epoetin-κ, for six months in total. The differences in the fluctuations between the clinical parameters prior to and following the change were subsequently examined. When examining the changes following the switch from epoetin-β to epoetin-κ, the patients were divided into three groups (9): N-N, L and H. Group N-N comprised patients whose hemoglobin (Hb) levels ranged between 10 and 12 g/dl prior to and following the change from epoetin-β to epoetin-κ; group L comprised patients whose Hb levels decreased to <10 g/dl at least once prior to the change to epoetin-κ; and group H comprised patients whose Hb levels increased to >12 g/dl at least once prior to the change to epoetin-κ. The frequency and dose of epoetin administration differed among the patients, with a dose of 750 units administered once a week in seven cases, twice a week in 14 cases and once every two weeks in one case; a dose of 1,500 units administered once a week in two cases and twice a week in five cases; and a dose of 3,000 units administered twice a week in one case (Table II).

## Results

As the most common dose among the case group, 750 units epoetin was administered once or twice a week and an examination of the changes of each average parameter was conducted, based on the average of the seven and 14 cases receiving this dose, respectively. The results of this analysis revealed that there was little fluctuation in the data prior to and following the switchover. It was concluded that the administration of ≥750 units of epoetin-β or epoetin-κ once or twice a week was appropriate from a clinical standpoint.

**Figure 1:**
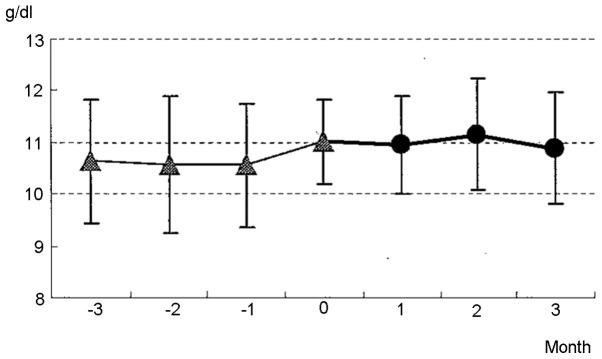
Changes in hemoglobin (Hb) levels prior to and following the switch from epoetin-β to epoetin-κ in patients injected with 750 units once or twice a week. Month 0: switch from epoetin-β to epoetin-κ.

**Figure 2:**
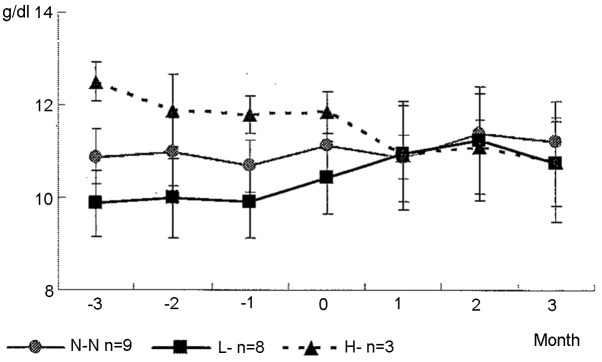
Changes in hemoglobin (Hb) levels prior to and following the switch from epoetin-β to epoetin-κ in patients injected with 750 units once or twice a week. The examination was conducted by dividing the patients into three groups. Group N-N, patients with Hb levels ranging between 10 and 12 g/dl prior to and subsequent to the change to epoetin-κ; Group L, patients with Hb levels that decreased to <10 g/dl at least once prior to the change to epoetin-κ; Group H, patients with Hb levels that increased to >12 g/dl at least once prior to switching over to epoetin-κ. Month 0: switch from epoetin-β to epoetin-κ.

**Figure 3:**
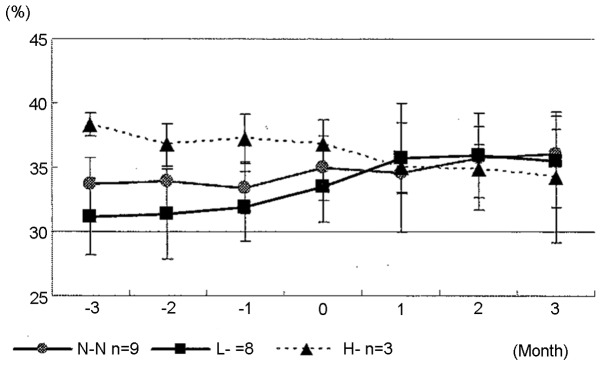
Changes in hematocrit prior to and following the switch from epoetin-β to epoetin-κ in patients injected with 750 units once or twice a week. The patients were divided into three groups. Group N-N, patients with Hb levels ranging between 10 and 12 g/dl prior to and subsequent to the change to epoetin-κ; Group L, patients with Hb levels that decreased to <10 g/dl at least once prior to the change to epoetin-κ; Group H, patients with Hb levels that increased to >12 g/dl at least once prior to switching over to epoetin-κ. Month 0: switch from epoetin-β to epoetin-κ.

**Figure 4:**
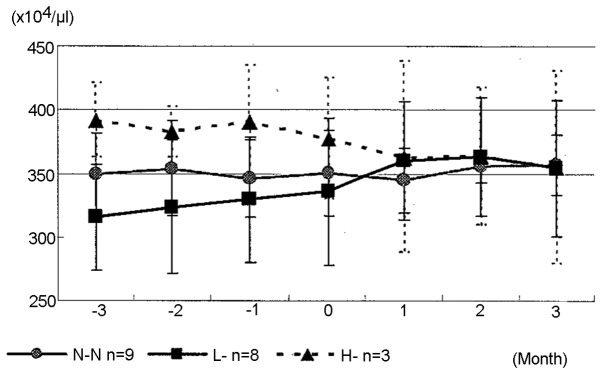
Changes in red blood cell count prior to and following the switch from epoetin-β to epoetin-κ in patients injected with 750 units once or twice a week. The patients were divided into three groups. Group N-N, patients with Hb levels ranging between 10 and 12 g/dl prior to and subsequent to the change to epoetin-κ; Group L, patients with Hb levels that decreased to <10 g/dl at least once prior to the change to epoetin-κ; Group H, patients with Hb levels that increased to >12 g/dl at least once prior to switching over to epoetin-κ. Month 0: switch from epoetin-β to epoetin-κ.

The changes in Hb levels are shown graphically in Fig. 1. Prior to switching to epoetin-κ, a reduction in the Hb levels was observed; however, the levels were predominantly stable over the period of observation. The changes in clinical parameters following the switch were also examined in the patient groups N-N, L and H. Following the change from epoetin-β to epoetin-κ, the Hb levels in each group remained at ~11 g/dl (Fig. 2), while the hematocrit (Ht) remained at ~35% (Fig. 3) and the red blood cell counts appeared to converge at 350×10^4^/μl (Fig. 4).

## Discussion

As standard formulations for the administration of <250 units of epoetin-β and -κ were not available in the present study, the anemia was controlled by decreasing the administration frequency of the drug. With regard to cases where 3,000 units of formulation are administered to maintain symptom control, it may be considered preferable to administer the drug twice a week at the same dose, rather than three times a week at a lower dose of 1,500 units, owing to the lower number of administrations per week.

The basic manufacturing patent for the materials used to manufacture rEPO expired in 2005 and, as such, there are no longer any restrictions on its development (5,6,11). Therefore, it is expected that rEPO will be manufactured with a high level of safety through processes that do not use animal byproducts. With regard to its usage and dosage, the treatment guidelines recommend that it be administered three times a week (9). However, following the enforcement of comprehensive coverage for the costs of dialysis treatment at medical facilities in 2006, it has been recommended that, in order to maintain the Hb standard value, the majority of patients may be expected to stably respond to an administration of 1,500 units three times a week or 3,000 units twice a week, while additional dosage restrictions are also expected (12). During phases II and III of its clinical trials, an evaluation was performed that involved switching from epoetin-α to this drug and, in this instance, the dosage was 1,500 or 3,000 units twice or three times a week (13). However, as it is expected that administrations at lower doses and lower frequencies are likely to be conducted in clinical settings, The Kan-Etsu Hospital has been proactively studying alternative dosages and has evaluated the equivalence of this drug with its predecessor drugs at dosages not used in phases II and III of its clinical trials. In a study conducted in 1998 (14), it was demonstrated that there was a correlation between the weekly rate of change in Hb levels and the frequency of adverse events. Furthermore, it was shown that the greater the degree of instability and the greater the decline, the higher the frequency of adverse events. In the study performed by Fishbane and Berns (15), the term ‘Hb Cycling’ was applied to the phenomenon in which fluctuations >1.5 g/dl in their degree of instability (observed for one year) repeated themselves in an eight-week cycle (15). In another study, Ebben *et al*(10) conducted a retrospective analysis of 150,000 patients receiving dialysis (10). Dividing the patients into three groups, ‘Target’, ‘High’ and ‘Low’, according to their Hb levels (similar to this experiment), the fluctuation patterns in Hb levels were observed. The results indicated that, irrespective of the time of occurrence, the proportion of cases included in the Target Group did not reach 50%.

The present study yielded results obtained during a six-month observation period, during which the administration of epoetin-β was changed to that of to epoetin-κ. Three months following the switch to epoetin-κ, the degree of instability had decreased. Although the situation subsequent to the change from epoetin-β to epoetin-κ requires further investigation, it may be concluded that the results obtained in this study are indicative of the clinical equivalence and the efficacy of epoetin-κ.

## Figures and Tables

**Table I:** General patient data.

Characteristic	Cases [n (%)]
Gender	
Male	26 (86.7)
Female	4 (13.3)
Age^a^ (years)	
<65	14 (46.7)
≥65	16 (53.3)
Origin	
Diabetes	8 (26.7)
Polycystic kidney	6 (20.0)
IgA nephritis	1 (3.3)
CGN	11 (36.7)
Other	4 (13.3)
HD term (years)	
<10	19 (63.3)
≥10	11 (36.7)

a Average age ± standard deviation, 63.0±11.3 years.

IgA, immunoglobulin A; CGN, chronic glomerulonephritis; HD, hemodialysis.

**Table II:** Injection frequency and dose of patients.

	Dose (IU)		
	
Frequency	750 (n)	1500 (n)	3000 (n)
Once a week	7	2	0
Twice a week	14	5	1
Every other week	1	0	0
